# Effect of TiO_2_ Nanoparticles on Capillary-Driven Flow in Water Nanofilters Based on Chitosan Cellulose and Polyvinylidene Fluoride Nanocomposites: A Theoretical Study

**DOI:** 10.3390/polym14142908

**Published:** 2022-07-17

**Authors:** Noureddine Mahdhi, Norah Salem Alsaiari, Abdelfattah Amari, Mohamed Ali Chakhoum

**Affiliations:** 1 Laboratory Materials Organizations and Properties, Tunis El Manar University, Tunis 2092, Tunisia; 2 Department of Chemistry, College of Science, Princess Nourah Bint Abdulrahman University, Riyadh 11671, Saudi Arabia; nsalsaiari@pnu.edu.sa; 3 Department of Chemical Engineering, College of Engineering, King Khalid University, Abha 61411, Saudi Arabia; 4 Research Laboratory of Processes, Energetics, Environment and Electrical Systems, National School of Engineers, Gabes University, Gabes 6072, Tunisia; 5 Laboratoire des Sciences de la Matière Condensée (LSMC), Université Oran 1 Ahmed Ben Bella, Oran 31100, Algeria; chakhoum.mohammed@edu.univ-oran1.dz

**Keywords:** nanofilter, nanocomposite, water, purification, capillary-driven flow, biocompatible, biodegradable

## Abstract

In this study, a novel concept of nanofiltration process of drinking water based on capillary-driven nanofiltration is demonstrated using a bio-based nanocomposites’ nanofilter as free power: a green and sustainable solution. Based on Lifshitz and Young–Laplace theories, we show that the chitosan (CS), cellulose acetate (CLA), and Polyvinylidene fluoride (PVDF) polymer matrixes demonstrate hydrophobic behavior, which leads to the draining of water from nanopores when negative capillary pressure is applied and consequently prevents the capillary-driven nanofiltration process. By incorporating 10%, 20%, and 30% volume fraction of titanium dioxide (TiO_2_) nanoparticles (NPs) to the polymers’ matrixes, we demonstrate a wetting conversion from hydrophobic to hydrophilic behavior of these polymer nanocomposites. Subsequently, the threshold volume fraction of the TiO_2_ NPs for the conversion from draining (hydrophobic) to filling (hydrophilic) by capillary pressure were found to be equal to 5.1%, 10.9%, and 13.9%, respectively, for CS/TiO_2_, CLA/TiO_2_, and PVDF/TiO_2_ nanocomposites. Then, we demonstrated the negligible effect of the gravity force on capillary rise as well as the capillary-driven flow for nanoscale pore size. For nanofilters with the same effective nanopore radius, porosity, pore shape factor, and tortuosity, results from the modified Lucas–Washburn model show that the capillary rise as well as the capillary-driven water volume increase with increased volume fraction of the TiO_2_ NPs for all nanocomposite nanofilter. Interestingly, the capillary-driven water volume was in range (5.26–6.39) L/h·m^2^ with 30% volume fraction of TiO_2_ NPs, which support our idea for capillary-driven nanofiltration as zero energy consumption nano-filtration process. Correspondingly, the biodegradable CS/TiO_2_ and CLA/TiO_2_ nanocomposites nanofilter demonstrate capillary-driven water volume higher, ~1.5 and ~1.2 times, respectively, more than the synthetic PVDF/TiO_2_ nanocomposite.

## 1. Introduction

The occurrence of micro-sized particle wastes such as heavy metals, microplatics, and pathogens in drinking water presents great risks for human health and biodiversity. Recently, Darren et al. performed water chemistry analysis and size fractionation sampling of drinking water at four houses in the city of Newark, New Jersey [1]. They found the existence of pyromorphite (Pb_5_(PO4)_3_Cl) microparticles with size < 100 nm in drinking water samples that passed through the tap or pitcher filtration units. Barbara et al. investigated 32 water bottle samples from 21 different brands of mineral water from Bavarian food stores [2]. They detected variable amounts of microsized plastics and pigments in both reusable and glass bottles. They ascribe their occurrence and possible contamination to various sources, such as from the washing machinery, the bottle cap, and other steps during the filling process. Additionally, other sources of drinking water (e.g., surface water, groundwater) are likely to be polluted by microparticle wastes such as heavy metals, pathogens, and pesticides [3,4,5].

Regarding this state-of-the-art, a supplementary domestic filtration process is an adequate solution for purification of the tap and bottled drinking water. Lately, a considerable literature has developed around the theme of the purification of drinking water from the nano- and micro-sized particles using reverse osmosis (RO), nanofiltration (NF), chemical coagulation, adsorption, and magnetic nanoparticles processes [6,7,8,9,10,11,12]. Typically, the Nanofiltration (NF) process is the suitable domestic purification process for tap and bottled drinking water. In fact, NF has greater flux and less energy use rates, operational under normal conditions, and its unique advantages of retaining at an optimum the essential multivalent and all monovalent minerals ions required for the human body [6,7,13,14]. However, the main common disadvantages of the previous processes are their energy consumption, removing all minerals essential ions required for human body (RO), and more difficult installation as a domestic purification solution. Moreover, the NF process commonly introduces supplementary pressure to drive water inside the nanopores and to increase the permeation rate of the nanofilter membranes, which leads to an increase in their cost-effectiveness [15,16].

Very recently, emerging studies focused on exploring the so-called capillary-driven nanofiltration process as a natural, free energy, and sustainable process for water purification [17,18,19,20]. This ubiquitous nanofiltration natural process allows water transport throughout nanoporous materials only using capillary pressure and without the help of external forces [19,20]. In fact, at the nanoscale, the flow of the water inside the nanoporous structure, known as imbibition, is driven by the capillary pressure. For hydrophobic materials, the capillary pressure tends to pull water from the nanopores. The reason for that is the nanofilter based on hydrophobic materials requires supplementary pressure driven to overcome the capillary pressure and to drive water inside the nanopores [21]. This fact may cause membrane destruction and then increase the cost-effectiveness of the NF process and loss in purification efficiency of the nanofilter membranes [15,16]. For hydrophilic materials, the water flows spontaneously throughout the nanostructure due to the capillary pressure and then generates a natural flow through the nanofilter without any additional pressure [17,18,19,20].

For instance, there are poor attempts to implant the capillary-driven nanofiltration using bio-based materials as a free energy, biocompatible, and sustainable solution for the purification of drinking water.

Therefore, the purpose of this study is to demonstrate how to develop a capillary-driven NF on bio-based porous nanocomposites as a newly sustainable domestic purification process of tap and bottled drinking water with zero energy consumption. To do so, it is essential to study quantitatively and qualitatively the effect nanofilter materials composition have on their wettability as well as their water permeability in terms of capillary-driven flow.

Actually, an emerging trend is underway to develop novel synthetic and biodegradable nanocomposite polymers for use in the NF process [22,23,24,25,26]. They suggest the incorporation of NPs-based metal oxides materials for increasing water permeability, mechanical strength, separation efficiency, and reducing fouling of the membrane [27,28,29].

In this paper, we aim to give a detailed theoretical investigation of the effect of NPs filling rates on the physical properties that involves the spontaneous capillary-driven NF: the surface energy, wettability, capillary pressure, and capillary-driven water volume for some biodegradable and synthetic nanocomposites commonly used as nanofilters for drinking water.

Titanium dioxide (TiO_2_) NPs was chosen as filler for the nanocomposites because of their prominent properties such as abundance in nature, biocompatibility, low-cost preparation with different sized and shaped particles (including nanowires, nanotubes, nanofibers, core-shell structures, and hollow nanostructures), and antiseptic and antibacterial properties [30]. Chitosan (CS) and cellulose acetate (CLA) were chosen as bio-based and biodegradable polymer matrix, whereas polyvinylidene fluoride (PVDF) was chosen as synthetic non-biodegradable polymer [31,32,33].

The following part of this study is dedicated to the presentation of the theoretical methods used for the evaluation of the surface energy, water contact angle, capillary pressure, and the capillary-driven water volume as function of the TiO_2_ NPs volume fraction. In the next section, we demonstrate that the pure polymer matrix exhibits a hydrophobic behavior, which leads to negative capillary pressure around the nanopores, that acts as draining force of water from the nanopores of the nanofilter and, therefore, prevents the capillary-driven NF process. Afterwards, the threshold filler rate of TiO_2_ NPs required to activate capillary-driven flow is determined for each polymer’s nanocomposite. Later, the enhancement of water flow throughout the nanocomposite’s nanofilters in terms of capillary rise and capillary-driven water volume will be demonstrated by increasing the volume fraction of the TiO_2_ NPs. At the end, using a recapitulative comparison study, we demonstrate that the nanofilter based on biodegradable CS/TiO_2_ and CLA/TiO_2_ nanocomposites provides better capillary-driven NF performance than the synthetic nanocomposites PVDF/TiO_2_ one.

## 2. Materials and Methods

### 2.1. Capillary Nanofiltration

#### 2.1.1. Nanofiltration and Capillary Pressure

**Figure 1:**
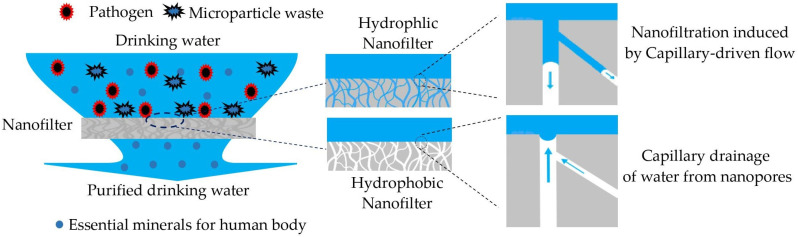
Schematic illustration of capillary-driven NF principle for r_p_ << λ_c_.

NF is purification process based on the flow of water through nanopores/nanochannel sizes from 1 to 10 nm for the removal of waste microparticles that have a diameter larger than the nanopores of the membrane [34]. Figure 1 shows a brief description of the principle of capillary-driven NF proposed in this study. The flow of water on both sides, superior and inferior, of the membrane is governed by gravity (from top to bottom). However, the flow of water throughout the NF membrane is driven by action of capillary pressure which depends on the wettability [17,35].

In fact, at normal conditions of temperature and pressure, the spontaneous flow of the water across porous structure is determined by an interplay of surface and gravity forces. The influence of the gravity force on the flow of water inside nanopore can be found by calculation of the so-called capillary length of water [36]:

(1) $$\lambda_{c}=\sqrt{\frac{\gamma }{\rho g}}$$

where γ and ρ are surface tension and the density of water and g is the acceleration of the gravity. For water (With γ = 72 10^−3^ N·m^−1^ and ρ = 1000 kg·m^−3^). Equation (1) yields a capillary length of water equal to 2.7 mm.

Usually, for surfaces with characteristic lengths smaller than λ_c_, the gravitational force can be neglected. Therefore, for nanofilter membrane with pore radius ranges from 1 nm to 10 nm, we have r_p_ << λ_c_ and then the influence of the gravity force in water flow is ignored. Consequently, the flow of water throughout the nanofilter membrane is a function of surface forces i.e., wettability and capillary pressure.

As presented in Figure 1, for the hydrophilic nanofilter membrane, the transport phenomenon of water throughout the nanopores is governed by so-called capillary-driven flow, whereas for a hydrophobic nanofilter membrane, the capillary forces pull water from the nanopore and this results in the drainage of water.

To evaluate the strength of the capillary pressure, we use the Young–Laplace equation, which relies on the capillary pressure to the contact angle of water θ, the surface tension of water γ_l_, and the capillary pore radius r_p_ by [37]:

(2) $$P_{c}=P_{\mathrm{nw}}-P_{w}=\frac{2\gamma_{l}\cdot \cos\theta }{r_{p}}$$

The capillary pressure (P_c_) acts as the difference between pressure non-wetting (P_nw_) and wetting (P_w_) phases.

**Figure 2:**
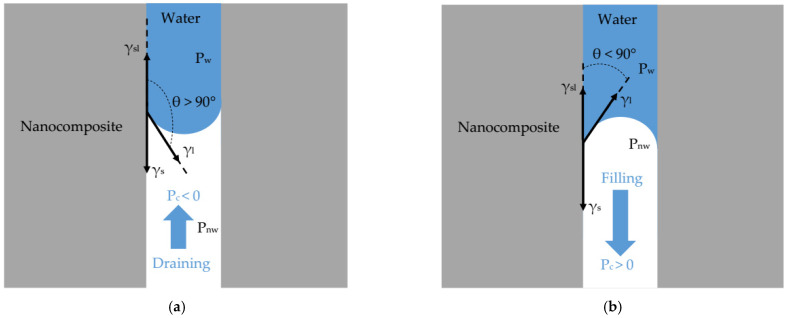
Capillary pressure action in nanopore r_p_ << λ_c_ (**a**) capillary filling with hydrophilic nanocomposite (**b**) capillary draining with hydrophobic nanocomposite.

As shown in Figure 2a, at the interface water–membrane–air, when the adhesion force (i.e., force with which water molecules adhere to a membrane surface) is less than the cohesion force of water molecules, the water does not penetrate spontaneously into nanopores and it forms a convex meniscus with contact angle above θ > 90°.

However, when the force of adhesion is greater than the cohesion force of water molecules and it forms a concave meniscus with contact angle less than 90° (Figure 2b), water molecules tend to penetrate spontaneously into nanopores [38]. This latter situation is defined as the capillary-driven flow known as spontaneous imbibition [17,18,19,35].

#### 2.1.2. Capillary Rise in Nanopore

Capillary rise is defined as the movement of water within porous materials due to the forces of adhesion, cohesion, and surface tension. In this study, we consider a straight nanopore of radius r_p_ << λ_c_ in contact with water of viscosity µ. For θ < 90°, the water is pulled inside the nanopore by the net interfacial force $2{\pi r}_{p}\gamma \cos\theta$. At the equilibrium, the final capillary rise y_f_ is determined by the force balance between the capillary force $f_{c}=2{\pi r}_{p}\gamma \cos\theta$ and the gravity force ($f_{g}={\rho g\pi r}_{p}^{2}y_{f}$) known as Jurin’s law [38]:

(3) $$y_{f}=\frac{2\gamma_{l}\cdot \cos\theta }{{\rho \mathrm{gr}}_{p}}$$

When y is much smaller than y_f_, the capillary force f_c_ and viscous friction force ($f_{f}=µ\cdot V\cdot y$) become the two drived forces which govern the flow of the water. This is the so-called viscous regime in which the capillary rise y as function of time t is described by Washburn’s law [39]:

(4) $$y\left(t\right)=\sqrt{\frac{r_{p}\gamma_{l}\cos\theta }{2\mu }t}$$

#### 2.1.3. Modified Lucas–Washburn for Predicting Capillary-Driven Water Volume

To take account of the morphological characteristics of nanoporous medium on capillary filling kinetics, Benavente et al. [40] developed the Lucas–Washburn (LW) model for predicting the capillary-driven liquid volume V over time t throughout nanoporous material, which is related to the capillary rise as in the following equation:

(5) $$V\left(t\right)=S\cdot \varphi \sqrt{\frac{\delta }{\tau }}\cdot y\left(t\right)=S\cdot \varphi \sqrt{\frac{{\delta r}_{p}\gamma_{l}\cos\theta }{2\mu \tau }t}$$

where S is the cross section of the nanofilter surface, φ is the porosity of the nanofilter membrane, δ is the pore shape factor (δ = 1 when the cross section of the pore is a perfect circle), τ is the correction factor of tortuosity (equal 1 to the straight pore channel and 1 < τ < 3 for random pore channels).

The tortuosity is directly related to the porosity within the classical fluid flow approach [41] as in the following equation:

(6) τ=1−0.77ln(φ)

#### 2.1.4. Wettability: Contact Angle and Surface Energy

Wettability plays a crucial role in determining water permeability of porous membrane. It is well-known that hydrophilic porous materials exhibit a higher permeability than hydrophobic ones [17]. According to relation 2, this evidence can be understood in terms of capillary pressure. In fact, the capillary pressure is positive (filling action) for hydrophilic materials and negative (draining action) for hydrophobic.

- Contact angle

According to Young’s equation, the contact angle θ (Figure 2) results from the thermodynamic equilibrium between the surface tension of water γ_l_, the interfacial tension water/nanocomposite γ_sl_, and the surface energy of the nanocomposites γ_s_ as [42]:

(7) $$\cos\theta =\frac{\gamma_{s}-\gamma_{\mathrm{sl}}}{\gamma_{l}}$$

where γ_sl_ is determined by [43] (p. 417):

(8) $$\gamma_{\mathrm{sl}}=\gamma_{s}+\gamma_{l}-2(\sqrt{\gamma_{l}^{D}\gamma_{s}^{D}}+\sqrt{\gamma_{l}^{P}\gamma_{s}^{P}}$$

where γ^D^ and γ^P^ denote the dispersive and polar component of surface energy.

- Surface energy

To evaluate the surface energy of nanocomposites as function of the TiO_2_ NPs volume fraction, we used a theoretical method that allowed calculating of the surface energy from the evaluation of the Hamaker constant as in the following equation [43] (p. 415):

(9) $$\gamma =\frac{H}{24\cdot \pi \cdot {\;x}_{0}^{2}}$$

where x_0_ the interatomic distance between two surfaces in contact called the cut-off distance typically equals to 0.165 nm and H is the Hamaker constant.

### 2.2. Method for Calculation of the Hamaker Constant

The Hamaker constant H is a fundamental constant for describing qualitatively and quantitatively the van der Waals (vdW) intermolecular interaction that governs the surface forces. On the basis of the Lifshitz theory, the Hamaker constant is function of the bulk properties of the interacting mediums: their dielectric constants ε, and their refractive indexes n [43] (p. 260):

(10) $$H=H^{P}+{\;H}^{D}$$

where H^P^ is the polar part of the Hamaker constant. It regroups the Keesom interaction that arises from permanent molecular dipoles and the Debye interaction that arises from permanent dipoles and induced dipoles [44,45]. It is expressed as a function as dielectric permittivity as in the following equation [43] (p. 260):

(11) $$H^{P}=\frac{3}{4}k_{B}T\;{\left(\frac{\varepsilon_{1}-\varepsilon_{2}}{\varepsilon_{1}+\varepsilon_{2}}\right)}^{2}$$

where k_B_ is the Boltzmann constant, T is the temperature, (ε_1_, ε_2_) are, respectively, the dielectric permittivity of the nanocomposite and air.

However, H^D^ is the dispersive part of the Hamaker constant H that regroups the London van der Waals forces resulting from the fluctuations in the charge densities of the electron clouds surrounding the nuclei of the atoms [46]. It is expressed as function of the refractive index of the three interacting mediums and it is given by the following equation [43] (p. 260):

(12) $$H^{D}=\frac{3{h\nu }_{e}}{16\sqrt{2}}\frac{{\left(n_{1}^{2}-n_{2}^{2}\right)}^{2}}{\;(n_{1}^{2}+n_{2}^{2})\sqrt{\left(n_{1}^{2}+n_{2}^{2}\right)}}$$

ν_e_ is the main electronic absorption frequency in the UV typically around 3 × 10^15^ s^−1^ [43] (p. 260), h is the Planck constant, and (n_1_, n_2_) are, respectively, the refractive index of nanocomposite and air in the visible spectrum (at 600 nm).

### 2.3. Model for Calculation the Dielectric Constant of Nanocomposites

The power-law model is used to calculate the dielectric constant as function of the volume fraction (Ф) of the NPs filler added to the nanocomposites. Hypothetically, we consider that the NPs filler have a spherical form and they are uniformly dispersed in the continuous polymer matrix [47]:

(13) $$\varepsilon_{c}^{1/3}={\Phi \varepsilon }_{m}^{1/3}+\left(1-\Phi \right){\;\varepsilon }_{f}^{1/3}$$

### 2.4. Models for Calculation of the Refractive Index

The refractive index of the nanocomposites is calculated using the popular mixing theory of Maxwell garnet [48]:

(14) $$n_{c}^{2}=n_{m}^{2}\frac{(n_{f}^{2}+2n_{m}^{2})+2\Phi \left(n_{f}^{2}-n_{m}^{2}\right)}{(n_{f}^{2}+2n_{m}^{2})-\Phi \left(n_{f}^{2}-n_{m}^{2}\right)}$$

where Φ is the volume fraction of NPs filler, n_c_, n_m_ and n_f_ are the refractive indexes, respectively, of nanocomposites, polymer matrix, and TiO_2_ NPs filler.

The method and models used in this study for evaluation of the surface energy have been validated by many pertinent research studies [43] (p. 278), [49,50,51,52].

### 2.5. Materials

The material chosen in this study are from two categories. Chitosan (CS) and cellulose acetate (CLA) were chosen as the bio-based and biodegradable polymer matrix. Whereas, polyvinylidene fluoride (PVDF) was chosen as synthetic polymer. These polymers have facilitated a great interest in drinking water and wastewater treatment using many processes, such as adsorption, NF, and reverse osmosis [22,23,24,25,26,27,28,29]. Their main advantageous properties are abundance, chemical stability in aqueous medium, mechanical strength, and cost-effectiveness [53,54,55].

However, TiO_2_ anatase NPs were chosen as filler to these polymer matrixes. Moreover, their common advantages with the polymer matrix, TiO_2_ NPs, provide many prominent properties for water treatment, such as biocompatibility, antibacterial. and good photostability [56,57].

Actually, there are many easy and ecofriendly techniques for manufacturing nanocomposites nanofilter membrane for water purification, such as the phase inversion process, thermally induced phase separation, chemical bond connection, and interfacial polymerization [58,59,60].

Hereafter, the volume fraction rates of TiO_2_ NPs added to CS, CLA and PVDF polymer matrix are fixed to 0%, 10%, 20%, and 30%, and they are designated in the study as presented in Table 1.

In Table 2, we summarized the dielectric constant and the refractive index of the materials for the calculation of the Hamaker constants.

## 3. Results and Discussion

All the calculations and the discussions of the results were made at standard temperature (T = 25 °C = 298.15 K) and pressure (P = 1 atm = 101.325 kPa).

### 3.1. Effect of TiO_2_ NPs Filling on Surface Energy

We can deduce from relation 2 the capillary pressure is function of the contact angle, the surface tension of water, and pore radius. Hence, we report the variation in the contact angle by varying the surface energy of nanocomposites during filling with TiO_2_ NPs and nanopore radius (1 to 10 nm), while the surface tension of water is always constant (γ_l_ = 72 10^−3^ J·m^−2^).

Table 3 shows an increase of the refractive index and the dielectric constant of all nanocomposites after incorporation TiO_2_ NPs filler. This raise is attributed to the elevated refractive index and dielectric constant of TiO_2_ NPs compared to those for the pure CS, CLA, and PVDF polymer matrixes.

Table 4 reports the Hamaker constants calculated from relations (10-11-12). It is apparent from this table that the Hamaker constants for the system nanocomposite–air are still positive and in the range of ×10^−20^ J [43] (p. 261). Interestingly, this suggests the preciseness of our used model for computing of the vdW intermolecular interactions that governs surface forces [43] (pp. 253–289).

After providing the Hamaker constant, the surface energy is then calculated using Equation (9). As it can be observed in Table 5 and Table 6, for purely CS, CLA, and PVDF polymer matrixes, the surface energy range values (20–43·10^−3^ J·m^−2^) are in good agreement with those reported by many studies (Table 6), which confirms the accuracy of our used model for the evaluation of the surface energy.

However, in accordance with our earlier observations on the Hamaker constant, the surface energy was, remarkably, increasing with filler rates (Φ = 10%, 20% and 30%) of TiO_2_ NPs for all nanocomposites. This increase was significantly important in the order PVDF/TiO_2_ < CLA/TiO_2_ < CS/TiO_2_. Therefore, it is well-known that the incorporation of TiO2 NPs give rise to an increase of the dispersion and polar vdW intermolecular interactions that governs surface forces of the nanocomposites.

To evaluate the changes in the intermolecular interactions at the interface nanocomposites-water after incorporation of the TiO_2_ NPs, the interfacial tension γ_sl_ was calculated using Equation (8) and summarized in Table 7. According to the observations on the surface energy, there was also an increase of the γ_sl_ with filler rates for all nanocomposites. In particular, for the same rate of Φ, we find that the γ_sl_ was more important in the order PVDF/TiO_2_-Water < CLA/TiO_2_-Water < CS/TiO_2_-Water.

Interestingly, during the incorporation of TiO_2_ NPs, the bio-based CS/TiO_2_ and CLA/TiO_2_ nanocomposites exhibit an improved γ_s_ as well as and γ_sl_ than for PVDF/TiO_2_ synthetic one.

### 3.2. Contact Angle and Capillary Pressure

As mentioned in Section 2.1.1, for nanopore with radius r_p_ (1–10 nm) < λ_c_ (water) = 2.7 mm, the effect gravitational force on the contact angle configuration is then neglected and only the intermolecular vdW surface forces contribute to the contact angle [36] (p. 36).

**Figure 3:**
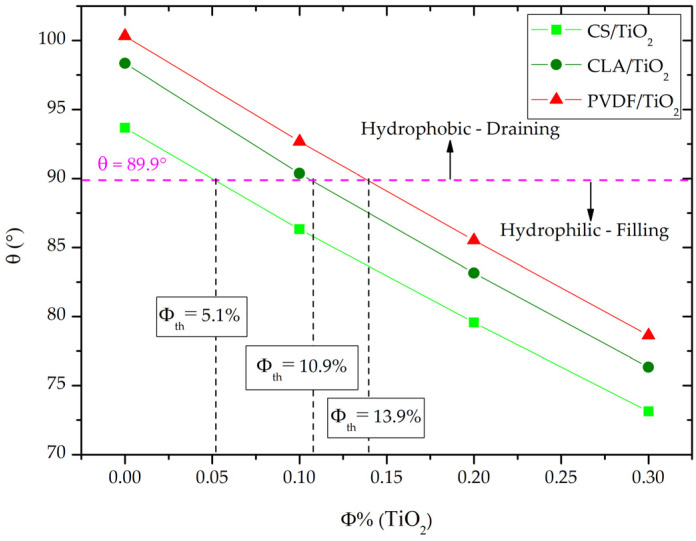
Water Contact angle of CS/TiO_2_, CLA/TiO_2_ and PVDF/TiO_2_ nanocomposites as function of volume fraction of TiO_2_ NPs.

To study the capillary pressure behavior of the nanocomposites, we first determined the contact angle at the three-phase boundary between nanocomposite–water–air using Young’s relation (relation 7). Figure 3 shows a decrease of the contact angle for all nanocomposites with increasing the volume fraction of TiO_2_ NPs. The CS, CLA and PVDF polymer matrix (Φ = 0%) exhibits a hydrophobic behavior with the water contact angle > 90°. Simultaneously, we can see from Table 5 and Table 7 for Φ = 0%, the interfacial tension γ_sl_ is greater than surface energy γ_s_ of polymer matrix which leads to the formation of a convex meniscus (P_c_ = P_nw_ − P_w_ < 0) at the interface water-air that prevent water to flow inside of the nanopore.

After incorporation of TiO_2_ NPs, a conversion from hydrophobic to hydrophilic character for CS/TiO_2_ nanocomposite is identified at Φ < 10%, while the hydrophobic character persists by filling with 10% < Φ < 20% for CLA/TiO_2_ and PVDF/TiO_2_ nanocomposites. Therefore, the CS/TiO_2_ nanocomposite provides enhanced hydrophilic behavior with low filling with TiO_2_ NPs than CLA/TiO_2_ and PVDF/TiO_2_ nanocomposites.

For depicting the changes in the contact angle on the capillary pressure, we calculated the evolution of the capillary pressure as function of the nanopore radius (1–10 nm) using relation 2. Then, we normalized the capillary pressure P_c_ to the atmospheric pressure (Pa = 101.325 kPa) to show the rate of changes caused by the incorporation of TiO_2_ NPs.

**Figure 4:**
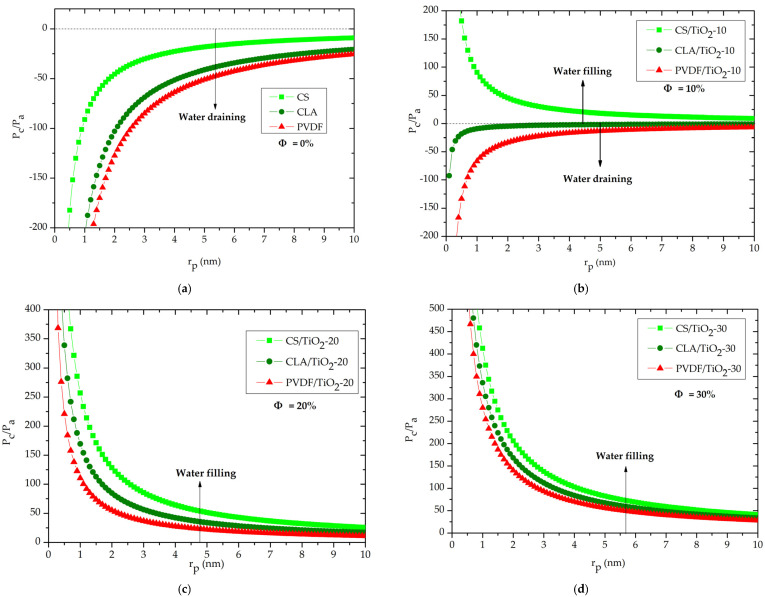
Capillary pressure as function of the pore radius for CS/TiO_2_, CLA/TiO_2_ and PVDF/TiO_2_: (**a**) Ф = 0%, (**b**) Ф = 10%, (**c**) Ф = 20%, (**d**) Ф = 30%.

For a pure polymer matrix (Figure 4a), the normalized capillary pressure was negative along the nanopore radius r_p_. As can be interpreted from relation 2, the negative value of P_c_ originates hydrophobic behavior of the CS, CLA and PVDF polymer matrix which leads to negative value of the term cosθ (Figure 3). Consequently, the meniscus is convex, and the capillary pressure tends to pull water from unwetted to wetted regions of the nanopore (Figure 2a), resulting in the drainage of water from the nanopore. In the other hand, such negative capillary pressure describes the necessary opposite pressure to provide rising water inside the nanopore in the case of the use of a nanofilter based on a purely polymer matrix. Therefore, for the CS, CLA, and PVDF polymeric nanofilter membranes, the NF process requires supplementary pressure with opposite and higher strength than the capillary pressure to squeeze water throughout the nanopore.

We now turn to study the capillary pressure after incorporation of the TiO_2_ NPs. As noted Figure 4b, the normalized capillary pressure for CS/TiO_2_-10 becomes positive which reveals a changing from draining to filling of water inside the nanopores. However, for CLA/TiO_2_-10 and PVDF/TiO_2_-10, the normalized capillary pressure becomes less important than for CLA and PVDF purely matrix but it still drains water from the nanopores. However, as demonstrated in Figure 4c,d for Φ = (20%, 30%), the capillary pressure becomes positive for all nanocomposites and still increases with increasing the volume fractions of TiO_2_ NPs.

**Figure 5:**
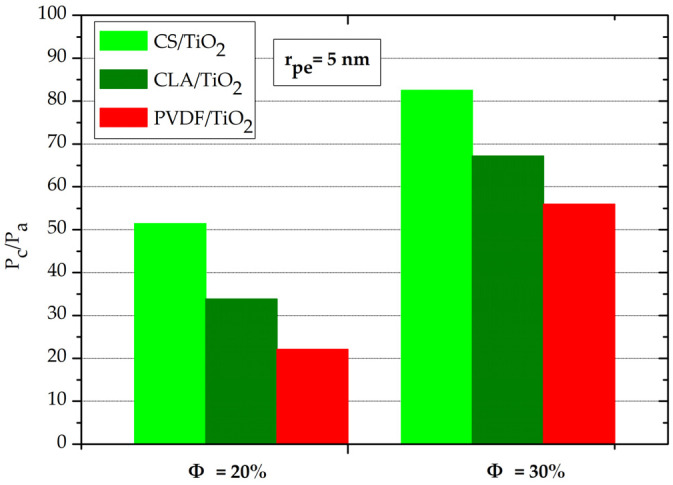
Normalized capillary pressure P_c_/P_a_ at r_pe_ = 5 nm and Φ = (20% and 30%) for CS/TiO_2_, CLA/TiO_2_ and PVDF/TiO_2_ nanocomposites.

Figure 5 depicts the relative evolution of the capillary pressure for Φ = (20%, 30%) at the same effective nanopore radius r_pe_ = 5 nm. For all nanocomposites, the P_c_/P_a_ (Φ = 30%) ≈ 2. P_c_/P_a_ (Φ = 20%). Interestingly, this demonstrates that the capillary pressure increases almost twofold with the addition rate of 10% (from 20% to 30%) of TiO_2_ NPs. In addition, it should be noted that the capillary pressure was more important in the order PVDF/TiO_2_ < CLA/TiO_2_ < CS/TiO_2_ nanocomposites.

Therefore, for future experimental valorizations of these finding, it is crucial to determine the threshold volume fraction that corresponds to conversion from draining to filling by capillary pressure. To do so, we determined the threshold of the volume fraction Φ_th_ of TiO_2_ NPs for what the contact angle equal 89.9°. Correspondingly, Φ_th_ at which there is a transition from P_c_ < 0 that is in favor of draining, to P_c_ > 0 that is in favor of capillary filling. As depicted in Figure 4, the Φ_th_ for CS/TiO_2_, CLA/TiO_2_ and PVDF/TiO_2_ were, respectively, 5.1%, 10.90%, and 13.90%. In Table 8, we summarized the corresponding threshold dielectric constant ε_th_, refractive n_th_, Hamaker constant H, the surface energy γ_th_ for CS/TiO_2_, CLA/TiO_2_, and PVDF/TiO_2_ nanocomposites.

### 3.3. Capillary Rise

We highlighted the effect of TiO_2_ NPs on surface energy, capillary pressure, and contact angle in the previous section. Now, we will explore the dynamics aspects of the filling of water by evaluation of capillary rise and volume uptake of water throughout the NF membrane for Ф > Ф_th_, i.e., when water flows inside the nanopore of the membrane under action of capillary pressure. Therefore, the capillary rise (y(t)) and water filling dynamics (V(t)) of water in nanocomposites were restrictively studied for CS/TiO_2_ at Ф = (10%, 20% and 30%) > Ф_th_ (CS/TiO_2_) = 5.1%, and for CLA/TiO_2_ and PVDF/TiO_2′_ at Ф = (20% and 30%) > Ф_th_ (PVDF/TiO_2_) > Ф_th_ (CLA/TiO_2_). For comparative purposes, they will juxtaposed with those and the y_th_(t) and V_th_(t) threshold ones.

**Figure 6:**
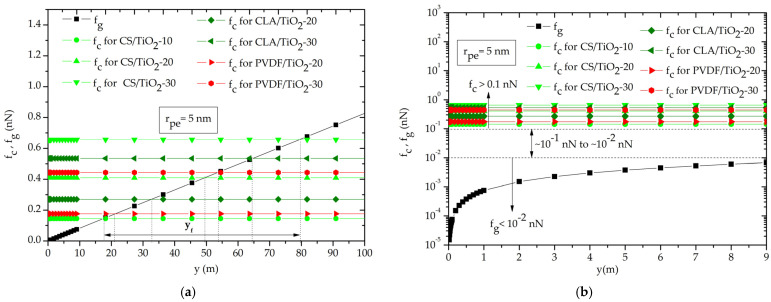
Gravity and capillary forces as function of distance rise in one nanopore: (**a**) the gravity force balance capillary force for at y_f_, (**b**) the strength of gravity force is negligible compared to the capillary force for distance (1 nm to 9 m).

To demonstrate the negligible effect of gravity in capillary filling kinetics, we show the gravity and capillary forces for each nanocomposite with effective nanopore radius r_pe_ = 5 nm. As it can be observed in Figure 6a, the capillary force balance with gravity force only for y_f_ that is over y > 10 m that is very higher to the ordinary dimension of the nanofilter. Incidentally, for a y < 10 m, the gravity force was less than capillary force by one order of magnitude f_g_ < 10^−1^·f_c_ (Figure 6b), which is in good agreement with previous studies [19] (p. 1624), [36] (p. 47). Consequently, for the here examined rise levels restricted by the maximum nanofilter height to less than 1 m, the contribution of the gravity force on the capillary filling kinetic of the water inside the nanopores is neglected (f_g_ << f_c_).

Therefore, the capillary rise is governed only by the balance between the viscous friction force (f_f_) and capillary force (f_c_). It is the so-called viscous regime, during which the capillary filling is well described by Washburn’s law (Section 2.1.2). Hereafter, to carry out the effect of the volume fraction on the capillary rise, we consider single capillary nanopore of uniform internal circular cross section with effective radius r_pe_ = 5 nm.

**Figure 7:**
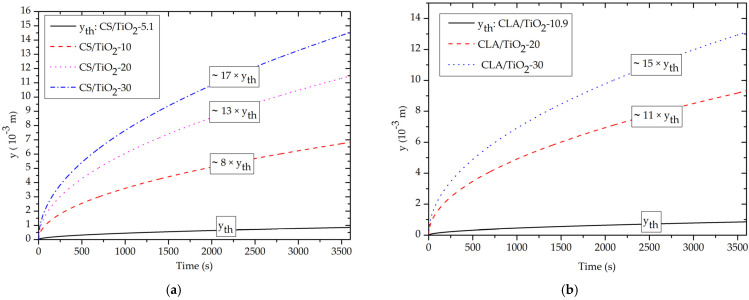

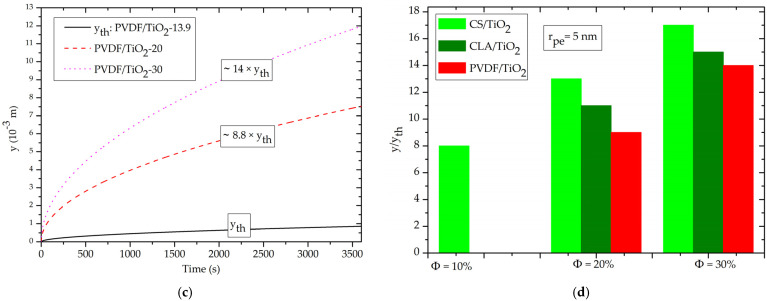
Effect of the volume fraction of TiO_2_ NPs on capillary rise y(t) with r_pe_ = 5 nm: (**a**) CS/TiO_2_, (**b**) CLA/TiO_2_, (**c**) PVDF/TiO_2_, (**d**) y/y_th_ as function of Ф of TiO_2_ NPs.

Figure 7 shows that the capillary rise y(t) increase with increasing volume fraction of TiO_2_ NPs. Obviously, the water rises spontaneously in nanopores under action of capillary pressure and without any other additional force for all nanocomposites. Interestingly, this result supports our idea for capillary-driven NF using nanoporous CS/TiO_2_, CLA/TiO_2_ and PVDF/TiO_2_ nanocomposites as nanofiltration process with zero energy consumption. Typically, the rise times in our study are in agreement with recent experimental and simulation studies [20,75]. In fact, for capillary nanopore, water rises slowly over time because the viscous friction forces become more important for nanoporous narrowed capillary, which reduces the overall capillary dynamics significantly.

In accordance with Section 3.2, for Ф = 10%, only the CS/TiO_2_-10 nanocomposite that exhibits a capillary rise (Figure 7d). Contrarily, for the CLA/TiO_2_-10 and PVDF/TiO_2_-10, the capillary pressure remains negative, which prevents the rise of water inside the nanopores. However, for Ф = (20% and 30%) (Figure 7a–c), all the nanocomposites exhibit spontaneous capillary rise. As expected from the study of capillary pressure, the capillary rise increases with volume fraction of the TiO_2_ NPs.

Figure 7d depicts the increases rates of the normalized capillary rise (y/y_th_) with volume fraction filler of TiO_2_ NPs. In particular, at Ф = 30%, the y/y_th_ were about ~17, ~15 and ~14, respectively, for CS/TiO_2_, CLA/TiO_2_ and PVDF/TiO_2_ nanofilter. Interestingly, this finding demonstrates that the biodegradable CS/TiO_2_ and CLA/TiO_2_ nanofilter exhibit higher capillary rise than the synthetic PVDF/TiO2 nanofilter. From a review of Table 3 and Table 5, we can thus reveal that the enhancement of the capillary rise can be made by increasing the refractive index of the based nanofilter materials.

### 3.4. Effect of Volume Fraction of TiO_2_ NPs on Capillary-Driven Water Volume

As detailed in Section 2.1.3, the modified LW model allows the calculation of the capillary-driven water volume throughout nanoporous membrane as a function of the morphological characteristics of the nanofilter, (φ, r_pe_, δ and τ), the physical properties of the water, (ρ, η, and γ_l_), and the contact angle θ which depends on the volume fraction of TiO_2_ NPs.

Therefore, to explore the effect of the incorporation of TiO_2_ NPs on the capillary-driven water volume, we consider that the morphological characteristics the nanocomposite nanofilter are the same for all nanocomposites. Hereafter, we consider the porosity of nanofilter φ = 0.7 with corresponding tortuosity τ = 1.27 (Relation 6) and non-circular cross section of nanopores with roundness δ = 0.5.

**Figure 8:**
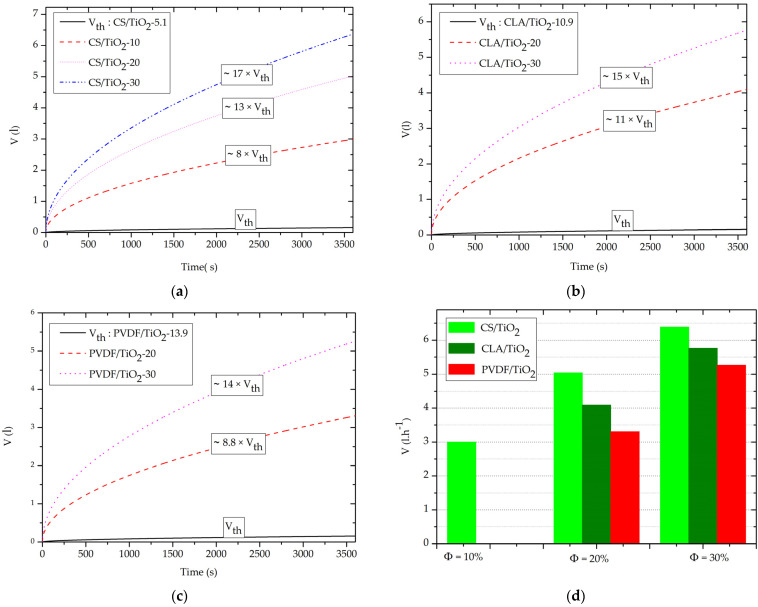
Evolution of the capillary-driven water volume V(t) for with S = 1 m^2^ and r_pe_ = 5 nm. (**a**) CS/TiO_2_, (**b**) CLA/TiO_2_, (**c**) PVDF/TiO_2_, (**d**) capillary-driven water flow rate (L·h^−1^) as function of the Ф of TiO_2_ NPs.

The capillary-driven water volume was calculated for surface nanofilter membrane S = 1 m^2^ and effective nanopore radius r_pe_ = 5 nm using relation 5. As shown in Figure 8a–c, for Ф = Ф_th_ (CS/TiO_2_-5.1 ≅ CLA/TiO_2_-10.9 ≅ PVDF/TiO_2_-13.9), the threshold capillary-driven water volume (V_th_) after one hour of filling was ~30 mL. In fact, at θ_th_ = 89.9°, the term cos(θ_th_) in Equation (5) is equal 1.7·10^−3^, which leads to a weak value of V_th_.

However, for Ф = 10% (Figure 8a–d), only the CS/TiO_2_-10 nanocomposites’ nanofilter exhibits a spontaneous capillary-driven nanofiltration with V ≈ 3 L·h^−1^. In agreement with capillary pressure (Section 3.2) and capillary rise (Section 3.3), this finding indicates the enhanced capillary-driven NF of CS/TiO_2_-10 nanocomposite in comparison with other CLA/TiO_2_-10 and PVDF/TiO_2_-10 nanocomposites, which do not demonstrate a capillary-driven flow at Ф = 10%.

While, for Ф = (20% and 30%), we clearly observe in Figure 8b-c-d a great increase of the capillary-driven water volume. In particular, CS/TiO_2_-30 has the highest capillary-driven nanofiltration with V = 6.39 L·h^−1^, followed by CLA/TiO_2_-30 with V = 5.76 L·h^−1^ and PVDF/TiO_2_-30 with V = 5.26 L·h^−1^. Accordingly, it is important to note that these orders of magnitude of capillary-driven water volume are directly in line with previous findings (2–25 L·h^−1^·m^−2^) [17,40,76]. This validates our theorical findings on the one hand, and motivate future experimental valorizations of this study on the other.

Interestingly, for the same incorporation rates Ф = (20% or 30%) of the TiO_2_ NPs, the increased V(t) of the CS/TiO_2_, in comparison to CLA/TiO_2_ and PVDF/TiO_2_ nanocomposites nanofilter, originate from the elevated surface energy of the CS polymer matrix (γ_s_ (CS) = 38.54 J·m^−2^) > (γ_s_ (CLA) = 31.47 J·m^−2^) > (γ_s_ (PVDF) = 26.26 J·m^−2^), which strongly proportional to the refractive index of the polymers matrixes (n (CS) = 1.53) > (n (CLA) = 1.47) > (n (PVDF) = 1.42). In addition, from study of the capillary-driven water volume, we demonstrate that all nanocomposites are prominent candidates for use as domestic supplementary purification of drinking water for removal of microparticle waste with zero energy consumption.

Crucially, the biodegradable CS/TiO_2_-30 and CLA/TiO_2_-30 nanocomposites nanofilter demonstrate capillary-driven water volume higher ~1.5 and ~1.2 times, respectively, more than the synthetic PVDF/TiO_2_-30 nanocomposite. In particular, CS/TiO_2_ nanocomposite offer the most improved capillary-driven NF behavior during the incorporation of the TiO_2_ NPs.

It is important to note that for the experimental validation, the nanocomposite materials do not have the same morphological characteristics (tortuosity, porosity, pore size, etc.). Therefore, the capillary-driven water volume calculated by the modified L-W model (relations 5 and 6) is directly proportional to the pore radius, shape factor, porosity, and cross section nanofilter, and inversely proportional to the tortuosity of the nanofilter materials.

The methodologies used along this study constitute simple and complete theoretical methods for prediction the wettability as well as the spontaneous capillary pressure and the capillary-driven flow for any material with known refractive index dielectric permittivity and physical properties of the fluids.

Overall, these findings constitute a quantitative and qualitative background for further experimental elaboration of capillary-driven nanofilter based on green and biodegradable CS/TiO_2_ and CLA/TiO_2_ nanocomposites.

## 4. Conclusions

In this study, from Lifshitz and Young-Laplace theories, it is shown that the polymer matrixes CS, CLA and PVDF provide hydrophobic contact angle (θ > 90°) and draining capillary pressure. Consequently, the pure polymers matrix does not perform a capillary-driven flow and then cannot be used as capillary-driven nanofilter for water purification.

However, after incorporation of TiO_2_ NPs, a wetting conversion from hydrophobic to hydrophilic were depicted for all CS/TiO_2_, CLA/TiO_2_ and PVDF/TiO_2_ with, respectively, threshold volume fraction TiO_2_ NPs for wetting conversion equal to 5.1%, 10.9%, and 13.9%.

For incorporation with Ф > Ф_th_, the contribution of gravity forces on capillary rise as well as the capillary-driven flow is neglected for the considered nanofilter with effective nanopore radius r_pe_ (~10 nm) < λ_c_ (water) =2.7 mm and with macroscopic size of nanofilter < 10 m.

For a single nanopore, an increase of capillary rise with increased volume fraction was demonstrated for all CS/TiO_2_, CLA/TiO_2_ and PVDF/TiO_2_ nanocomposites. However, for the same incorporation rates of TiO_2_, the biodegradable CS/TiO_2_ and CLA/TiO_2_ exhibits a higher capillary rise than PVDF/TiO_2_. This increase was attributed to the higher surface energy proportional to the refractive index of the dielectric permittivity. Consequently, this fact reveled that water rise sin nanopores without the help of any external force for all nanoporous nanocomposites. This important finding supported our proposed idea for capillary-driven nanofiltration as zero energy consumption nanofiltration process.

Based on the modified L-W model, we demonstrated that capillary-driven water volume for CS/TiO_2_, CLA/TiO_2_, and PVDF/TiO_2_ nanocomposites increases with increasing TiO_2_ NPs volume fraction. Interestingly, at 30% volume fraction of TiO_2_ NPs, the biodegradable CS/TiO_2_ and CLA/TiO_2_ nanocomposites nanofilter demonstrate capillary-driven water volume higher by ~1.5 and ~1.2 times, respectively; more than the synthetic PVDF/TiO_2_ nanocomposite.

Collectively, CS/TiO_2_ and CLA/TiO_2_ biodegradable nanocomposites were the better candidates for purification of drinking water by capillary-driven nanofiltration process as sustainable, environmentally safe, and zero energy consumption.

## Figures and Tables

**Table 1:** Affected names of CS, CLA, and PVDF filled TiO_2_ NPs nanocomposites.

Φ% of TiO_2_ NPs	Nanocomposite		
	CS/TiO_2_	CLA/TiO_2_	PVDF/TiO_2_
0	CS	CLA	PVDF
10	CS/TiO_2_-10	CLA/TiO_2_-10	PVDF/TiO_2_-10
20	CS/TiO_2_-20	CLA/TiO_2_-20	PVDF/TiO_2_-20
30	CS/TiO_2_-30	CLA/TiO_2_-30	PVDF/TiO_2_-30

**Table 2:** Dielectric constants and refractive indexes of polymers matrixes, TiO_2_ NPs filler, water, and air at room temperature (298.15 K).

Material		ε at 1 MHz	n at 600 nm
Bio-based polymer	CS	5.5 [61]	1.53 [62]
	CLA	5 [63]	1.47 [64]
Synthetic polymer	PVDF	8.5 [65]	1.42 [66]
Filler	TiO_2_ (Anatase)	86 [67]	2.60 [67]
Air		1 [43]	1 [43]
Water		78.4 [68]	1.33 [68]

**Table 3:** Refractive index and dielectric constant of CS/TiO_2_, CLA/TiO_2_ and PVDF/TiO_2_ nanocomposites as function of volume fraction of TiO_2_ NPs.

Φ% (TiO_2_)	n			ε		
	CS/TiO_2_	CLA/TiO_2_	PVDF/TiO_2_	CS/TiO_2_	CLA/TiO_2_	PVDF/TiO_2_
0	1.53	1.47	1.42	5.5	5	8.5
10	1.61	1.56	1.51	8.36	7.76	11.82
20	1.70	1.65	1.61	12.08	11.40	15.91
30	1.80	1.75	1.70	16.77	16.02	20.86

**Table 4:** Hamaker constants of CS/TiO_2_, CLA/TiO_2_ and PVDF/TiO_2_ nanocomposites as function of volume fraction of TiO_2_ NPs.

Φ% (TiO_2_)	CS/TiO_2_			CLA/TiO_2_			PVDF/TiO_2_		
	H^P^ (10^−21^ J)	H^D^ (10^−20^ J)	H (10^−20^ J)	H^P^ (10^−21^ J)	H^D^ (10^−20^ J)	H (10^−20^ J)	H^P^ (10^−21^ J)	H^D^ (10^−20^ J)	H (10^−20^ J)
0	2.08	5.11	5.325	2.00	4.62	4.82	2.37	4.19	4.42
10	2.36	5.81	6.049	2.32	5.36	5.59	2.53	4.98	5.23
20	2.54	6.48	6.738	2.52	6.09	6.34	2.65	5.75	6.01
30	2.670	7.13	7.399	2.65	6.78	7.04	2.73	6.48	6.75

**Table 5:** Surface energy of CS/TiO_2_, CLA/TiO_2_ and PVDF/TiO_2_ nanocomposites as function of volume fraction of TiO_2_ NPs.

Φ% (TiO_2_)	CS/TiO_2_			CLA/TiO_2_			PVDF/TiO_2_		
	$\gamma_{s}^{P}$ $\left(10^{-3}\;J\cdot m^{-2}\right)$	$\gamma_{s}^{D}$ $\left(10^{-3}\;J\cdot m^{-2}\right)$	$\gamma_{s}$ $\left(10^{-3}\;J\cdot m^{-2}\right)$	$\gamma_{s}^{P}$ $\left(10^{-3}\;J\cdot m^{-2}\right)$	$\gamma_{s}^{D}$ $\left(10^{-3}\;J\cdot m^{-2}\right)$	$\gamma_{s}$ $\left(10^{-3}\;J\cdot m^{-2}\right)$	$\gamma_{s}^{P}$ $\left(10^{-3}\;J\cdot m^{-2}\right)$	$\gamma_{s}^{D}$ $\left(10^{-3}\;J\cdot m^{-2}\right)$	$\gamma_{s}$ $\left(10^{-3}\;J\cdot m^{-2}\right)$
0	0.72	37.82	38.54	0.66	30.80	31.47	0.93	25.33	26.26
10	0.93	48.91	49.84	0.89	41.68	42.57	1.07	35.87	36.94
20	1.08	61.04	62.12	1.05	53.78	54.84	1.17	47.83	49.00
30	1.18	74.15	75.33	1.17	67.05	68.22	1.24	61.16	62.40

**Table 6:** Surface energy of CS, CLA and PVDF polymer matrixes provided by this study and from literature.

Polymer	$\gamma_{s}\;\left(10^{-3}\;J\cdot m^{-2}\right)$	
	This Study	Literature
CS	38.54	34–43 [69,70]
CLA	31.47	30–34 [71,72]
PVDF	26.26	22–29 [73,74]

**Table 7:** Interfacial tension of CS/TiO_2_, CLA/TiO_2_ and PVDF/TiO_2_ with water as function Φ of TiO_2_ NPs.

Φ% (TiO_2_)	γ_sl_ (10^−3^ J·m^−2^)		
	CS/TiO_2_	CLA/TiO_2_	PVDF/TiO_2_
0	43.16	41.92	39.18
10	45.23	43.04	40.32
20	49.09	46.25	43.40
30	54.43	51.20	48.22

**Table 8:** Corresponding threshold ε_th_, n_th_, H and γ_th_ for θ = 89.9° of CS/TiO_2_, CLA/TiO_2_ and PVDF/TiO_2_ nanocomposites.

Nanocomposite	CS/TiO_2_	CLA/TiO_2_	PVDF/TiO_2_
Ф_th_ (%)	5.10	10.90	13.90
n_th_	1.57	1.57	1.55
ε_th_	6.83	7.92	13.24
H (10^−20^ J)	9.08	8.97	8.55
γ_th_ (10^−3^ J·m^−2^)	43.83	42.62	41.51

## Data Availability

Not applicable.
